# Assessing the value of artificial intelligence-based image analysis for pre-operative surgical planning of neck dissections and iENE detection in head and neck cancer patients

**DOI:** 10.1007/s12672-025-02798-4

**Published:** 2025-05-30

**Authors:** Benedikt Schmidl, Cosima C. Hoch, Robert Walter, Markus Wirth, Barbara Wollenberg, Timon Hussain

**Affiliations:** 1 Department of Otolaryngology Head and Neck Surgery, Technical University Munich, Munich, Germany; 2 Department of Diagnostic and Interventional Radiology, Technical University Munich, Munich, Germany; 3 Department of Otolaryngology Head and Neck Surgery, RWTH Aachen University, Aachen, Germany

**Keywords:** Image recognition, LNM, ChatGPT, Artificial intelligence

## Abstract

**Objectives:**

Accurate preoperative detection and analysis of lymph node metastasis (LNM) in head and neck squamous cell carcinoma (HNSCC) is essential for the surgical planning and execution of a neck dissection and may directly affect the morbidity and prognosis of patients. Additionally, predicting extranodal extension (ENE) using pre-operative imaging could be particularly valuable in oropharyngeal HPV-positive squamous cell carcinoma, enabling more accurate patient counseling, allowing the decision to favor primary chemoradiotherapy over immediate neck dissection when appropriate. Currently, radiological images are evaluated by radiologists and head and neck oncologists; and automated image interpretation is not part of the current standard of care. Therefore, the value of preoperative image recognition by artificial intelligence (AI) with the large language model (LLM) ChatGPT-4 V was evaluated in this exploratory study based on neck computed tomography (CT) images of HNSCC patients with cervical LNM, and corresponding images without LNM. The objective of this study was to firstly assess the preoperative rater accuracy by comparing clinician assessments of imaging-detected extranodal extension (iENE) and the extent of neck dissection to AI predictions, and secondly to evaluate the pathology-based accuracy by comparing AI predictions to final histopathological outcomes.

**Materials and methods:**

45 preoperative CT scans were retrospectively analyzed in this study: 15 cases in which a selective neck dissection (sND) was performed, 15 cases with ensuing radical neck dissection (mrND), and 15 cases without LNM (sND). Of note, image analysis was based on three single images provided to both ChatGPT-4 V and the head and neck surgeons as reviewers. Final pathological characteristics were available in all cases as HNSCC patients had undergone surgery. ChatGPT-4 V was tasked with providing the extent of LNM in the preoperative CT scans and with providing a recommendation for the extent of neck dissection and the detection of iENE. The diagnostic performance of ChatGPT-4 V was reviewed independently by two head and neck surgeons with its accuracy, sensitivity, and specificity being assessed.

**Results:**

In this study, ChatGPT-4 V reached a sensitivity of 100% and a specificity of 34.09% in identifying the need for a radical neck dissection based on neck CT images. The sensitivity and specificity of detecting iENE was 100% and 34.15%, respectively. Both human reviewers achieved higher specificity. Notably, ChatGPT-4 V also recommended a mrND and detected iENE on CT images without any cervical LNM.

**Discussion:**

In this exploratory study of 45 preoperative CT Neck scans before a neck dissection, ChatGPT-4 V substantially overestimated the degree and severity of lymph node metastasis in head and neck cancer. While these results suggest that ChatGPT-4 V may not yet be a tool providing added value for surgical planning in head and neck cancer, the unparalleled speed of analysis and well-founded reasoning provided suggests that AI tools may provide added value in the future.

## Introduction

Head and neck squamous cell carcinoma (HNSCC) is a heterogenous disease with a lack of validated preoperative and prognostic biomarkers [1]. Depending on the subsite, lymph node metastasis (LNM) is common, and one of the few early prognostic factors. An important characteristic of LNM is extranodal extension (ENE), which is associated with a significantly worse prognosis. While ENE does not necessarily determine the type or extent of neck dissection performed, it is often considered an indication for adjuvant chemotherapy [2, 3]. While positron emission tomography (PET)/computed tomography (CT) imaging increases the preoperative diagnostic sensitivity [4], there is still a lack of accuracy of preoperative imaging modalities in LNM of HNSCC [5]. Another aspect is that a significant percentage of HNSCC are resected with close or insufficient margins or too aggressively [6], due to insufficient pre- and perioperative imaging, leading to a compromised prognosis of patients. Next to the histopathological analysis of a biopsy, imaging is the primary diagnostic tool, also for the evaluation of recurrent or persistent disease [7], and for evaluating the extent of therapy in the form of surgical resection or radiotherapy. Imaging is also the basis for the removal of cervical lymph nodes as recommended for cases with LNM, and due to the probability of occult metastases of 20% also as an elective treatment of the neck. While in the past radical neck dissection including major vessels, muscles and nerves, was recommended, radical neck dissection is nowadays reserved only for cases with tumor infiltration of these structures [8]. The most common form of neck dissection is now the selective neck dissection (sND), removing only nodal groups at risk for metastasis [9]. Accurate preoperative planning is essential for the surgical treatment of patients with HNSCC, with improved imaging modalities, as well as improved image interpretation [10, 11]. Predicting ENE using pre-operative imaging is particularly valuable in oropharyngeal HPV-positive squamous cell carcinoma (OPSCC), which enables more accurate patient counseling, allowing the decision to favor primary chemoradiotherapy over immediate neck dissection. This approach could help avoid triple modality treatment, which often leads to increased morbidity [12, 13]. 

Computer-aided diagnosis (CAD) systems are one of the most recent developments of image analysis and were introduced to automate image interpretation and increase the efficiency [14, 15]. Using these systems, the existing workflows can be enhanced and streamlined [16]. Additional benefits are pattern recognition abilities and the optimization of resources by reducing the need for manual review [17]. Deep neural networks could thereby improve the diagnostic capability of algorithms to levels of human expertise [18]. At the same time the use of these models is limited by the need for extensive and specific training and customization [15, 19].

OpenAI recently introduced the extension ChatGPT-4 V for the existing large language model (LLM) ChatGPT 4.0, which enables the LLM to analyze images and examine visual content, in addition to the original strengths of advanced natural language processing (NLP) and contextual understanding [20]. While there are only a few studies investigating image interpretation with ChatGPT-4 V yet, LLMs can in principle access large datasets including image data in seconds and are therefore able to collect and summarize information of studies and other clinical data [21]. This way, the summarization of the information of a CT image by an LLM might become the basis of clinical decision making of oncological cases in the future [22–24]. The interpretation of clinical data of HNSCC patients was discussed in recent studies [25, 26]. Preoperative imaging of head and neck cancer was not evaluated by LLMs so far, even though the advancements of the latest LLM suggest that ChatGPT-4 V could potentially enhance the efficiency of CT scan interpretation and offers significant benefits compared to manual image interpretation.

The objectives of this study were therefore to firstly assess the preoperative rater accuracy by comparing clinician assessments of ENE and the extent of neck dissection to AI predictions, and secondly to evaluate the pathology-based accuracy by comparing AI predictions to final histopathological outcomes. Additionally, the study evaluated the time efficiency of AI, as well as its ability to detect and characterize lymph node metastasis for accurate preoperative staging.

## Materials and methods

### Patient cohort

This study comprised a total of 30 randomly selected CT Neck scans depicting LNM of HNSCC patients and 15 scans of patients with a CT scan without LNM (as a control group) at the Department of Otorhinolaryngology/Head and Neck Surgery. Clinical and histological tumor characteristics were collected from the electronic patient file and multidisciplinary tumor board (MDT) documents at the time of diagnosis. Out of the 30 LNM CT scans, 15 patients had received a modified-radical neck dissection (mrND), while 15 patients had a selective neck dissection (sND). In total 5 out of 15 patients in the sND group had pathological ENE, compared to 9 out of 15 patients in the mrND group. There was no case of occult metastasis in the 15 cases of eND. The gold standard for comparison was the known (histological) diagnosis and radiological interpretation. Exclusion criteria were the presence of foreign material resulting in artifacts and secondary malignancy. To ensure patient confidentiality, the data were anonymized before being shared with the researchers, making patient identification impossible. This study was approved by the ethics committee of the Technical University of Munich (Reference: 2024-184_1-S-NP). Approval for the waiver of informed consent was obtained from the Ethics Committee of the Technical University of Munich. The characteristics of the patient cohort are depicted in the Supplementary Table 1.

### Artificial intelligence/image recognition by ChatGPT prompt formatting and data evaluation

Since ChatGPT-4 V only interprets images of certain data formats [27], currently there is no option to analyze whole CT datasets in the DICOM format. At the same time, only a limited number of images can be analyzed in one prompt. For that reason, an experienced head and neck radiologist was tasked with analyzing the whole CT datasets and selecting 3 scans (one axial, one sagittal and one coronal) for each patient that depicted the LNM with the largest probability of vessel infiltration. If the radiologist suggested that there was a LNM that does not have an anatomical relationship to the large vessels, scans showing the largest diameter of the LNM were selected. For CT datasets showing no LNM, with a subsequent eND, scans were chosen that depict the large vessels on the side of the malignancy to allow the best comparison of scans. These scans were then subsequently converted into the JPEG format prior to submission into ChatGPT-4 V.

Prior studies revealed that next to the image interpretation alone, prompt formatting in ChatGPT-4 V is important for receiving convincing responses [28]. The contextual relationship between the image and the prompt in the user's query seems to be essential for LLMs to formulate a response [29]. For this reason, in the preliminary phase of this study the prompt design was iterated several times to achieve the final prompt. The iterations were varied with regard to of the amount of information. Two independent reviewers assessed the responses of each prompt and the resulting responses with the final evaluation matrix. The final prompt version reached the highest total score and provided the most consistent and accurate result. That final prompt was used for all of the 45 cases in the evaluation phase. The final version of the prompt in this study resembled the prompt of a study by Dehdab et al. [30] and Shifai et al. [29]: “For research purposes only and with the understanding that no clinical decisions will be made based on this AI-generated interpretation, examine the provided neck images from a single patient with an HNSCC. Identify any notable radiographic features in a descriptive manner. Is there extranodal extension or infiltration and what would be the extent of a subsequent neck dissection? This AI-generated interpretation will be subsequently reviewed by qualified experts for accuracy and educational value”.

Each prompt was used three times to get the most consistent answer and to minimize variability. The differences in the responses between those three different runs were minor. There was no further interaction with the LLM. The LLMs prompt history was erased, and the next case was presented, to prevent any influence from previous responses.

All of the responses were gathered and evaluated using a double-blinded method. All reviewers independently scored the answers to mitigate subjective biases. Each response was evaluated by two independent experienced medical experts in head and neck cancer. The grading scales for Summarization, Clinical Recommendation, and Explanation, by Sorin et al. [31] were used for a qualitative assessment, whereas the postoperative histopathological report after resection of the LNM was compared to the preoperative interpretation by ChatGPT-4 V.

**Fig. 1:**
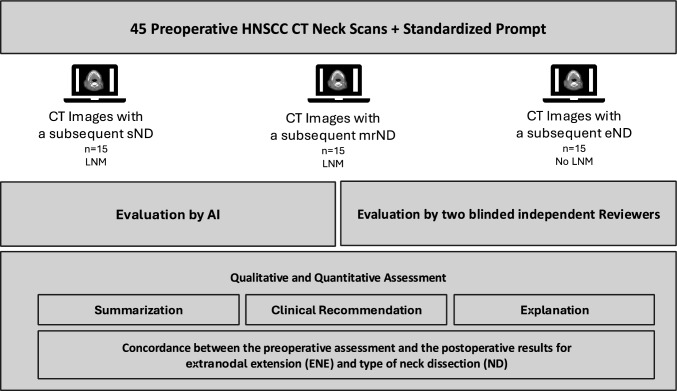
Depiction of the preoperative CT scans before a mrND, sND or eND and the corresponding interpretation by ChatGPT-4V. The sagittal, coronal and axial images are depicted. The answers were copied from the response page

In the next step for the 15 cases with mrND another prompt was added “Analyze the CT images to identify anatomical features, abnormalities, or markers that could indicate a higher risk of surgical complications. Based on these observations, assess the risk level for complications and provide an explanation for each identified risk factor. Additionally, if applicable, compare these features to common findings associated with elevated surgical risk in similar cases” to assess the identification of important structures and risk stratification for these cases. The study design is shown in the flowchart in Fig. 1. The datasets generated during and analysed during the study are available from the corresponding author on reasonable request. All methods were carried out in accordance with relevant national and international guidelines and regulations. The study used publicly available, anonymized clinical data. No identifiable human data were used, and all analyses adhered to established standards for secondary data use in medical research.

### Statistical analysis

Cohen's kappa coefficient was used to measure inter-rater reliability. Standard performance metrics (sensitivity, specificity, positive predictive value, and negative predictive value) were calculated. The Mann–Whitney U test was used to calculate significant differences between the rating of the responses of the LLM. P-values were adjusted with the Bonferroni correction method for multiple testing. A p-value of less than 0.05 was considered statistically significant.

## Results

### Preoperative CT scan analysis and ChatGPT-4 V response structure

**Fig. 2:**
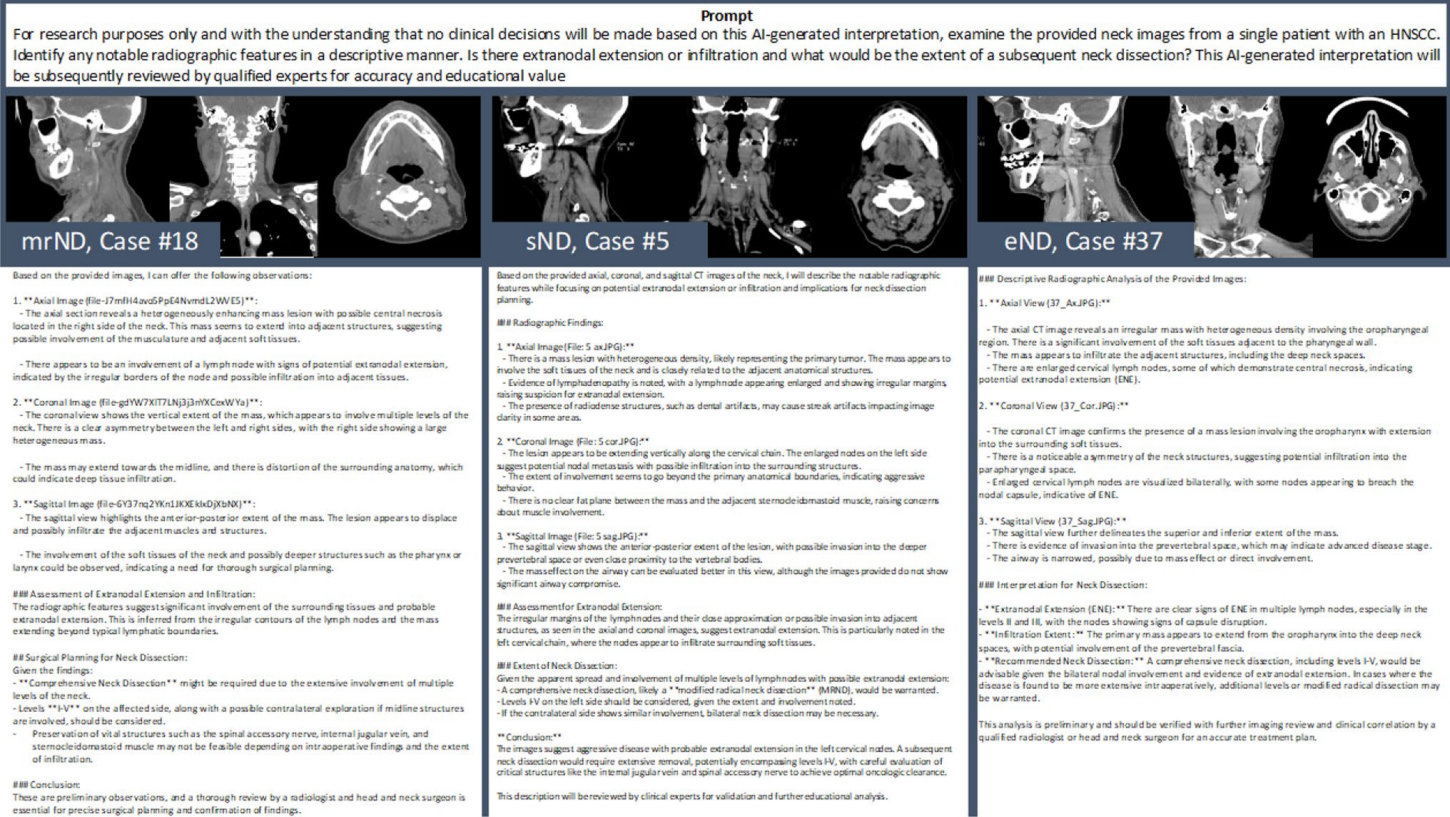
The standardized prompt and exemplary responses of ChatGPT-4V with the preoperative CT images for three cases (mrND, sND, eND). The responses of ChatGPT-4V were copied and presented to the independent blinded reviewers for evaluation. At the same time the CT images were also presented to the reviewers for evaluation as a comparison. *eND* elective neck dissection, *sND* selective neck dissection, *mrND* modified-radical neck dissection

In total 45 preoperative Neck CT scans were analyzed by ChatGPT-4 V. The prompt that was used for this study was carefully selected in a preliminary prompt design step that involved rigorous testing and iterating of prompts, until the responses generated by the final version of the prompt reached the highest rating in the performance matrix of this study. That prompt was used for the subsequent evaluation phase, in which the images of CT Neck scans that were selected by an experienced radiologist were analyzed by ChatGPT-4 V. A standardized response of ChatGPT for the different case scenarios of a sND, a mrND and an eND is depicted in Fig. 2.

ChatGPT-4 V began each response with an introductory phrase, followed by the section “Radiographic Findings”, in which the AI tool interpreted the CT scan of the Neck, analyzed the image and specified the scan including the general appearance and pathologies. The prompt was answered for each image separately and each scan was analyzed separately. ChatGPT-4 V recognized the correct plane of the scans in all of the cases and highlighted the presence of LNM and the involvement of critical structures such as the prevertebral fascia or relation to the carotid artery. Artifacts were also noted. Even though the project focused on the LNM and the majority of image sets therefore did not depict the primary tumor, ChatGPT-4 V tried to identify a potential primary mass in many cases. Surprisingly also the involvement of the airway was described for most of the cases even though the prompt did not specifically ask for this information. The response was for example “The mass appears to be infiltrating anteriorly towards the prevertebral fascia. There is significant encroachment on the airway structures, which could complicate surgical management”. The next section of the response was the extent of imaging-detected ENE (iENE), in which ChatGPT-4 V suggested a potential ENE based on the irregular nodal margins, the general appearance of the LNM or the involvement of adjacent structures. ChatGPT-4 V was able to recognize the importance of potential iENE for the staging, surgical planning, and the prognosis of the patient. Corresponding to the prompt there was a separate section of the response of almost all cases termed “Neck Dissection Considerations” or “Surgical Planning for Neck Dissection” This section was quite heterogeneous, since ChatGPT-4 V sometimes highlighted the exact lymph node levels that needed to be resected in the neck dissection based on the images, whereas in other cases there was only a general description and broad recommendation, similar to the majority of radiological reports. Nonetheless ChatGPT-4 V listed critical structures such as the spinal accessory nerve, internal jugular vein, and sternocleidomastoid muscle. For each case there was a short discussion of the potential types of neck dissection and the implications in terms of surgical removal of structures such as the jugular vein. Especially in the cases in which a subsequent mrND was performed, limitations of surgery due to involvement of critical structures were highlighted. As a final verdict ChatGPT told the user at the end of the response of every case either that “This interpretation is based on visual assessment only and should be correlated with clinical findings and further expert radiological review”, or that “This interpretation is purely educational and should not be used for clinical decision-making. A thorough review by qualified radiologists and clinicians is essential”. ChatGPT also recommended additional clinical examinations, such as clinical and endoscopic evaluation, or even further imaging modalities. Exemplary CT images and the responses of ChatGPT for a case with a subsequent mrND, a case with a sND and a case with an eND are depicted in Fig. 2.

On average ChatGPT-4 V required 31.2 s to respond to the prompt. This response time was not compared with the time needed by the two reviewers, as the reviewers were not asked to provide a structured report in this study.

### Comparative accuracy of clinicians and AI and performance against histopathological outcomes

**Fig. 3:**
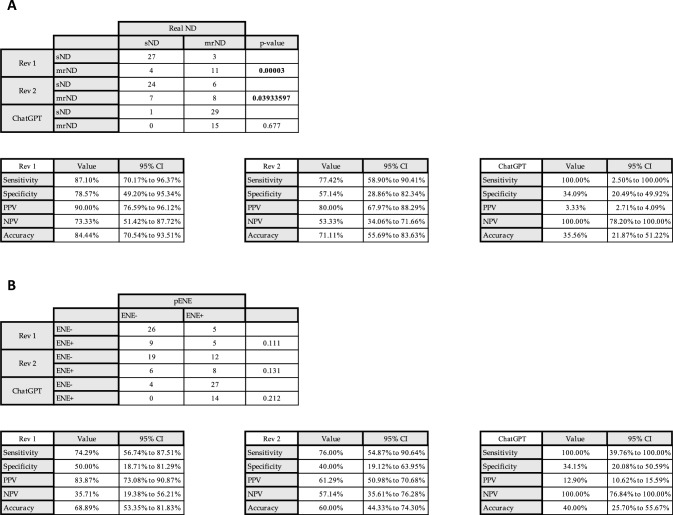
Analysis of the image interpretation and evaluation of neck dissection and iENE in comparison to the reviewers. **A** Comparison of the prediction of two independent reviewers for the type of a subsequent neck dissection based on the CT images. **B** Comparison of the prediction of two independent reviewers for the iENE based on the CT images. *Rev 1* Reviewer 1, *Rev* Reviewer 2. Fishers Exact Test was used to calculate the statistical significance

For assessing the preoperative rater accuracy by comparing clinician assessments of the extent of neck dissection to AI predictions, ChatGPT-4 V recommended a mrND for 97.8% of the cases in total (44 out of 45). In the cohort that was analyzed, 15 patients underwent a mrND due to tumor invasion of the jugular vein, sternocleidomastoid muscle or even skin. Out of these 15 cases, ChatGPT-4 V recommended a mrND for 100% of the cases, compared to 96.7% for the cases that actually underwent an eND or sND (29 out of 30). In contrast, in the evaluation matrix the two independent head and neck surgeons had to decide whether the patient was a candidate for a mrND based solely on the three retrospective CT images that were interpreted by ChatGPT-4 V. The surgeons recommended a radical neck dissection for in total 33.3% of the cases (15 out of 45, with a sensitivity of 87.1% and a specificity of 78.6% for Reviewer 1 and a sensitivity of 77.4% and a specificity of 57.1% for Reviewer 2. ChatGPT-4 V demonstrated a sensitivity of 100%, and a specificity of 34.1%. The positive predictive value and negative predictive value were 3.3% and 100.0%, with an overall accuracy of 35.6% (Fig. 3). For assessing the preoperative rater accuracy by comparing clinician assessments of iENE to AI predictions, in the next step, the two reviewers evaluated whether there was iENE in the preoperative CT images. This resulted in the interpretation of 31.1% of the cases as iENE positive (14 out of 45), compared to ChatGPT identifying iENE in 91.1% of the image sets (41 out of 45 cases), with a sensitivity of 100% and a specificity of 34.2%. The positive predictive value and negative predictive value were 12.9% and 100.0%, with an overall accuracy of 40.0%. The two reviewers achieved a sensitivity of 74.3%, a specificity of 50.0%, another sensitivity of 76.0% and a specificity of 40.0% (Fig. 3).

**Fig. 4:**
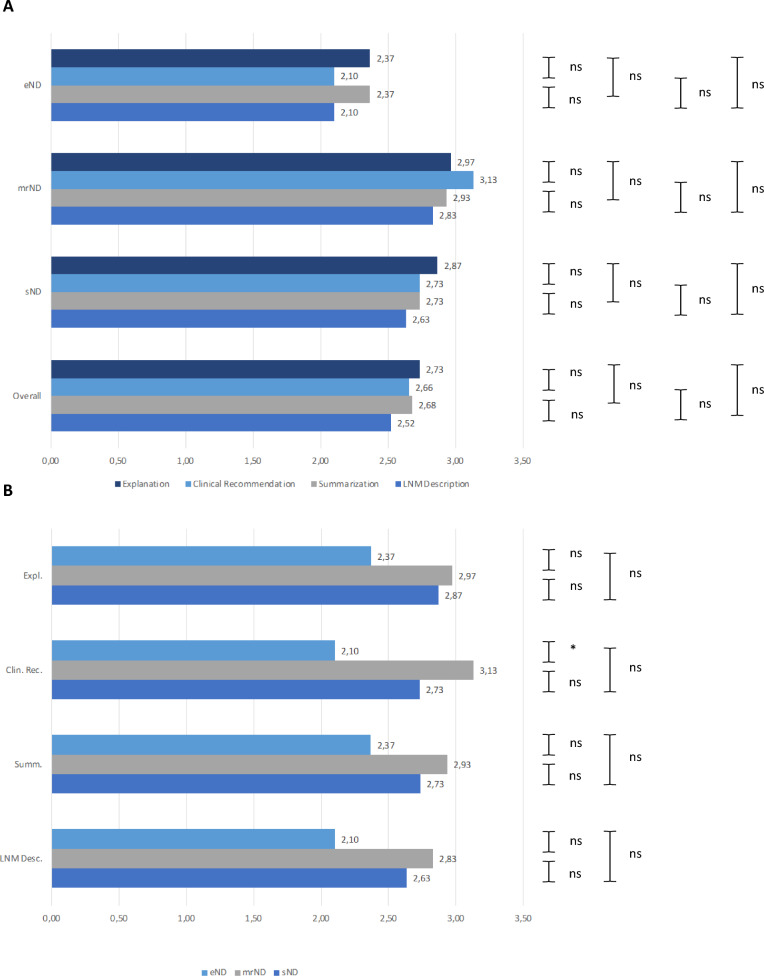
Overall rating of the qualitative performance of preoperative image interpretation by ChatGPT 4.0. **A** Comparison of the grading of summarization of text, clinical recommendation, explanation on the image interpretation of ChatGPT-4V made by two independent reviewers for CT images of a subsequent selective neck dissection (sND), elective neck dissection (eND), modified-radical neck dissection (mrND). **B** Comparison for the detailed categories of Summarization, Explanation and Clinical Recommendation. Each bar is the average of the two independent reviewers grading. # = number. * equals p<0.05, ** equals p<0.01, *** equals p<0.001. *Expl.* Explanation, *Clin. Rec.* Clinical Recommendation, *Summ*. Summarization, *Desc*. Description

The subjective evaluation of ChatGPT-4 V’s performance focused on its description of LNM, assessed in terms of agreement among reviewers. Additionally, its ability to summarize information, provide clinical recommendations, and offer explanations was rated across different cohorts. For summarization a total score of 2.68 was reached, compared to 2.93 in the mrND cohort, 2.73 in the sND cohort, and 2.37 in the eND cohort. For clinical recommendation a total score of 2.66 was reached, compared to 3.13 in the mrND cohort, 2.73 in the sND cohort, and 2.10 in the eND cohort. For explanation a total score of 2.73 was reached, compared to 2.97 in the mrND cohort, 2.87 in the sND cohort, and 2.37 in the eND cohort (Fig. 4A, B). There was a significantly better result for the rating of clinical recommendation in the mrND cohort compared to the eND cohort (p = 0.03). A two-way ANOVA reached a statistically significant p-value of the group effect of 7.80 × 10^−8^ and a nonsignificant Category Effect (p = 0.513) and Interaction Effect (p = 0.885). Cohen's kappa coefficient was used to measure inter-rater reliability and reached substantial to fair agreement based on the category (Supplementary Table). This analysis highlights variations in ChatGPT-4 V’s subjective performance across the different groups, particularly in clinical recommendations, where its effectiveness varied significantly.

### Risk stratification and surgical planning considerations

In the next phase of the study, the 15 cases of mrND were again subjected to the analysis of ChatGPT-4 V to assess the identification of essential structures and risk stratification for these cases. ChatGPT-4 V prioritized vascular structures as the most critical risk factor, followed by soft tissue involvement, bone and cartilage invasion, and airway infiltration. Additional high-risk factors identified included calcified vascular structures, deep-seated lymph node metastases (LNM), and proximity to major nerves. Based on these structures ChatGPT-4 V also gave a risk assessment for 73.3% (11 out of 15) of the mrND cases ranging from elevated to moderately to moderately high to high to extreme risk. One case (9.9%) was deemed with an elevated risk, two cases with moderate risk (18.2%), 6 cases with moderately high risk (54.5%) and two cases with high risk (18.2%). There were no cases in the extreme category in this study. The two high risk cases were both ENE positive and parts of the sternocleidomastoid muscle had to be resected in addition to the jugular vein. It was always stated that in cases with extensive LNM the surgical risks are greatly increased, and a multidisciplinary approach is recommended. At the same time ChatGPT was not able to rank the risk of each morbidity based on the likelihood.

## Discussion

This study represents the first evaluation of the use of the AI-based image interpretation as a preoperative tool for assessing lymph node metastasis (LNM) and guiding surgical decision-making in patients with head and neck squamous cell carcinoma (HNSCC). The primary objectives were twofold: Assessing rater accuracy by comparing clinician evaluations of LNM description and extranodal extension (ENE) to AI-generated predictions, thereby examining ChatGPT-4 V’s ability to match expert judgment in detecting key radiological features. Secondly, evaluating pathology-based accuracy by comparing AI-predicted LNM characteristics and surgical recommendations to final histopathological findings, assessing the reliability of ChatGPT-4 V in preoperative planning in a cohort of 45 patients with a HNSCC who subsequently underwent neck dissection. The AI-generated responses were rigorously evaluated by two independent, blinded head and neck surgeons, while the CT images were carefully selected by an experienced radiologist. By exploring the potential and limitations of ChatGPT-4 V in preoperative CT scan interpretation, this study provides valuable insights into its feasibility as a supplementary diagnostic tool in head and neck oncology.

Since AI-based image interpretation tools promise an enhanced performance in terms of speed, efficiency and improved diagnostic accuracy, while also reducing costs in healthcare, radiologists and head and neck surgeons are investigating innovative ways to use the most recent AI tools [32, 33]. One these potential applications is the use of AI for the preoperative analysis of CT Neck scans for surgical planning of neck dissection. A neck dissection is a highly variable surgical procedure that necessitates accurate preoperative planning in the form of imaging to detect the infiltration of essential structures and the extent of resection [34], as a more radical approach such as a mrND results in a high postoperative morbidity [35]. Accurate preoperative imaging interpretation might reduce this morbidity but is compromised by technical limitations and the sensitivity and specificity of conventional imaging [5]. While ChatGPT-4 V was originally designed for NLP [36–41], the processing and interpretation of images was recently added and turned out to be exceptionally fast in this study with a processing time from prompt to response of only 30.21 s per case. Other CAD programs already improved the efficiency of radiologists, by reducing CT reading times by 7–75% [19, 42], but the interpretation still took significantly longer (several minutes) compared to the results of previously published studies [43, 44].

In terms of the overall image interpretation, ChatGPT-4 V demonstrated a structured way of radiological reporting, highlighting general structures at risk during a neck dissection, and was able to calculate a risk-score. There was a significantly better result for the rating of clinical recommendation in the mrND cohort compared to the eND cohort, and overall, the results for CT images of a subsequent mrND were rated better than eND or sND images. When asked about the details of the image interpretation, such as specific structures of the neck or the probability of functional postoperative impairments, ChatGPT-4 V was not able to provide this information. At the same time the responses of ChatGPT-4 V showed various limitations. In this study, a mrND was recommended for almost all cases, compared to the significantly lower rate of 33.3% by the two head and neck surgeons as reviewers which was close to the rate of actually performed mrND. While both reviewers differed in their interpretation of the CT images, they achieved significantly better results than ChatGPT in detecting cases with LNM resulting in a mrND. Consistently, ChatGPT-4 V tended to recommend a more radical approach than the two human reviewers, even for CT images without any visible LNM. The prompt itself may have contributed to this result, since ChatGPT-4 V is at its core still a LLM and therefore its answers are strongly based on the textual relationship of the prompt [45]. Hence, even in patients without LNM, the textual information in the prompt suggesting cervical LNM, may lead the model interpret any asymmetry as potential LNM. This challenge may arise from factors such as inflammation, post-treatment effects, or anatomical variations, which alter the appearance of lymph nodes and may result in AI overinterpretation [46]. Additionally, potential dataset biases, such as an overrepresentation of high-risk cases, may have influenced the AI’s decision-making.

CT scans of the Neck are also highly sensitive to artefacts due to movement of the patient, dental work and deformity of soft tissue [47], resulting in asymmetries of structures. The differentiation of physiological tissue and LNM is complex, since both present with similar attenuation values on CT images, even though the criteria of ill-defined borders, infiltrative growth patterns, or a mass formation in CT Neck scans are suspicious for LNM [48]. Nevertheless, from a medical perspective, it seems interesting and promising that ChatGPT-4 V seems to be over-cautious rather than negligent as suggested by the high sensitivity in this study, albeit accompanied by very low specificity levels. This suggests an intrinsic bias of the AI model since a mRND was recommended for a large majority of cases, including those that had undergone eND. The reason for this bias cannot be investigated currently since it is not possible ot access the training data, but more aggressive surgical approaches might have been overrepresented. While the conservative approach of ChatGPT may prioritize oncologic safety, it does not always align with clinical decision-making aimed at balancing oncological control with functional outcomes. At the same time, the NPV of 100% and low PPV of 12.9% observed in this study highlight that while the model is highly effective at ruling out metastasis, it lacks reliability in confirming positive cases, which could result in false positives and unnecessary clinical or diagnostic interventions. This emphasizes that ChatGPT-4 V has limited value in surgical decision-making at this stage. Future refinements of the AI model need focus on training the model with a more diverse dataset to improve its ability to distinguish between appropriate levels of neck dissection. Expert-guided fine-tuning using diverse, well-annotated datasets could also enhance the model’s ability to differentiate between benign and malignant findings more accurately. Additionally, iterative model adjustments and validation in prospective studies will be necessary to optimize AI-driven recommendations for preoperative planning.

Since this is the first study using AI to analyze preoperative CT Neck scans for the surgical planning of neck dissections, the results in terms of sensitivity and specificity can only be compared to studies on different applications of image interpretation of ChatGPT-4 V. One of these studies investigated the use of ChatGPT-4 V for analyzing CT scans of NSCLC patients. Here, the model achieved a specificity of 60.47% and a sensitivity of 27.27% in the detection of NSCLC [30], while a recent study reported a sensitivity of 64%, and specificity of 30% in detecting abnormalities from CT scans of the liver [49]. Another exploratory study evaluated the potential for making an accurate diagnosis of melanoma based on photographic images, achieving a sensitivity of 78% and specificity of 46% to differentiate benign and malignant lesions [29]. The first meta-analysis of the general use of ChatGPT-4 V in radiology revealed, that 84.1% (37 out of 44) of studies demonstrated ChatGPT's effectiveness, but none suggested unsupervised use in clinical practice [50]. Even though there are multiple studies investigating CAD for detecting lung nodules in CT scans [51, 52], there are only a few studies using AI for imaging of the neck. The focus of these studies is mostly on segmentation of head and neck anatomy for the planning of radiotherapy [53–55]. Similar to the results of our study, AI tools are able to aid the detection of head and neck malignancy in imaging, as seen in a study of MRI and positron emission tomography (PET)/CT imaging of recurrent nasopharyngeal carcinoma reaching a sensitivity of 79.5% and specificity of 91% [56]. Another study of the detection of intracranial aneurysm reached a sensitivity of 84.5% and specificity of 18.2%, with the additional finding that AI-assistance could increase the diagnostic performance of a junior to the level of a senior physician, while even senior physicians improved significantly [57]. Compared to the results of this study, ChatGPT-4 V demonstrated similar results for the interpretation of CT lung scans, probably since these CT scans also depict the complex vascular and bronchial networks of the lung and comorbidities such as obstructive pulmonary disease or pneumonia [58].

In our study, the value of ChatGPT-4 V for iENE in CT Neck scans was also evaluated and compared to the assessment of two independent reviewers. iENE can be visualized on pre-treatment morphologic imaging and was proposed as a surrogate for pathological ENE, defined as extension of tumor cells outside the LN capsule into the perinodal soft tissue on histopathologic examination [59]. This could enable more accurate patient counseling, allowing the therapy recommendation to favor primary chemoradiotherapy over surgical approaches by minimizing treatment-related complications and avoiding triple modality treatment [12, 13, 60].

Recently an international group of head and neck radiologists generated consensus recommendations and proposed iENE to assist in guiding treatment selection for HNSCC, while at the same time highlighting the unresolved challenges including criteria to best diagnose iENE and the use of associated terminology [61]. ENE is an important prognostic factor and was added to the eighth edition of the American Joint Committee on Cancer (AJCC) and the Union for International Cancer Control (UICC) TNM classification [62]. In this study ChatGPT-4 V was not able to reliably identify iENE. The sensitivity of the reviewers in our study in detecting iENE was similar and ranged between 77.4% and 87.1%. However, and of note, there was no statistically significant association between iENE and pathological ENE, neither by the iENE of the reviewers, nor by the iENE of ChatGPT-4 V. As already seen for the interpretation of the neck dissection, ChatGPT-4 V achieved a high sensitivity by stating that signs of iENE are visible in the CT images but did not reach a high specificity and was therefore not able to stratify patients.

ChatGPT-4 V in its current form has currently limited value in surgical planning due to its challenges in accurately interpreting radiological features. Unlike deep learning models such as convolutional neural networks (CNNs), which are specifically trained on large, annotated datasets for image recognition, ChatGPT-4 V primarily relies on language-based reasoning and lacks direct image-processing capabilities optimized for radiological assessment [63]. At the same time CNNs lack the ability to process or understand unstructured textual data or integrate diverse multimodal inputs and are therefore outperformed by LLMs in some studies [64, 65]. Future advancements involve hybrid AI models that integrate CNN-based image analysis with language models to enhance both image interpretation and contextual understanding [63, 66]. Additionally, some studies incorporate AI-driven image segmentation, feature extraction, feature labelling and multimodal data—including clinical and pathological information—which may improve diagnostic accuracy and decision-making relevance [66–68].

While this study highlights the potential of AI for interpreting head and neck imaging, and the rapidly evolving landscape of AI-based image recognition will enable future applications, there are currently several limitations to consider prior to implementing AI into the clinical practice. An important limitation of the current landscape of LLMs, including the use of ChatGPT-4 V in this study, is the importance of the prompt design. Different prompts can yield varying responses, potentially influencing study results [28]. To make up for this, multiple prompts were tested in this study in an iterative prompt design phase, and two reviewers selected the prompt that reached the highest score of the final evaluation matrix. Early versions of the prompt resulted in inconsistent responses and did not reach a convincing result in the evaluation matrix. This highlights the need for standardized prompt strategies, including clear descriptions and a high specificity and to ensure consistency. This concern was also raised by several researchers, with methods of standardization being investigated to reduce bias [25, 38, 69]. These strategies were applied for the prompt design of this study and involved a high specificity, clear description of the setting, and thorough testing and iteration [70]. Future research may explore tailored prompts for specific clinical situations, such as CT image interpretation of recurrence or distant metastasis.

Another major limitation of the current use of ChatGPT-4 V for CT image interpretation is that only a limited number of single images of CT Neck scans could be investigated, since the software currently does not allow the analysis of a whole DICOM set. This limits the number of images that can be used in the prompt and also result in the loss of imaging data due to compression or conversion of images [71]. In this study each image of a scan was obtained by an experienced radiologist after an assessment whole DICOM set to determine the slices that best represented the LNM and a potential vascular infiltration. The quality of each scan was reviewed before submission to ChatGPT-4 V. While this technical limitation accounts for some of the limited accuracy achieved by ChatGPT-4 V’s analysis, both human reviewers were also provided with single images, hereby leveling the playing field. On the other hand, the subjective selection of imaging slices by the head and neck radiologist, introduces a potential bias in the data fed into the AI model. While this approach reflects real-world clinical decision-making, it inherently lacks the fully automated, objective analysis that other approaches, such as voxel-based AI models can provide [72]. Voxel-based deep learning methods have been successfully implemented in other studies and offer the potential for a more comprehensive, three-dimensional evaluation of tumor and nodal characteristics [73, 74]. However, such approaches require substantial computational resources, standardized imaging protocols, and extensive annotated datasets [75, 76] Future research should explore the feasibility of voxel-based AI models to enhance automation and reduce subjectivity in ENE prediction. Another limitation is the heterogenous distribution of ENE in our cohort. Since pathological ENE is more common in cases resulting in a mrND, collecting a homogenous cohort of the same rate of ENE in all groups is difficult and the heterogeneity of the ENE might have influenced the findings of this study [2, 9].

Other limitations of this study include the monocentric approach of analyzing the CT scans of patients of only one European institution. This preliminary study analyzed only 45 cases, and future research should involve larger cohorts and needs to include imaging that depicts recurrent disease, more heterogeneous imaging modalities, and a broader range of clinical scenarios to further evaluate the utility and accuracy of AI-based assessments in diverse patient populations. Some studies also suggest that the responses of LLMs depend on the source material and training data, resulting in the need to investigate multicentric/multiethnic data [77]. At the same time ChatGPT-4 V as a public tool will enable these studies by with its global accessibility [78, 79].

There is also the “black box phenomenon”, describing the lack of transparency of the way information is analyzed by AI [80]. Without knowing which information was the basis of the final result in this study, the translation of findings into the real world is limited [36–41]. While guidelines and clinical studies are the backbone of clinical decision making, there is no access to the source information of the LLM, including imaging information, resulting in the inability to validate and reproduce the results of studies [20, 79, 81].

Altogether, ChatGPT-4 V demonstrated potential in assisting with CT Neck scan interpretation for the preoperative analysis of neck dissection but does not yet add clinical value. The human reviewers in this study achieved a higher degree of accuracy when assessing the preoperative CT images and for predicting the type of subsequent neck dissection compared to ChatGPT-4 V. For iENE the results of this study were similar. Further advancements in AI and machine learning are necessary to provide true clinical value, the rather cautious approach by the model seems encouraging.

## Conclusions

The study is the first assessing the potential of artificial intelligence-based preoperative CT Neck scan interpretation by ChatGPT-4 V in head and neck cancer. Overall, the model’s assessment was highly sensitive to adverse features but very limited in specificity, therefore not yet adding clinical value.

## Data Availability

The datasets generated during and analysed during the study are available from the corresponding author on reasonable request.
